# C-Nail versus plate osteosynthesis in displaced intra-articular calcaneal fractures—a comparative retrospective study

**DOI:** 10.1186/s13018-021-02349-x

**Published:** 2021-03-20

**Authors:** Eva Steinhausen, Wolfgang Martin, Rolf Lefering, Sven Lundin, Martin Glombitza, Bastian Mester, Nikolaus Brinkmann, Marcel Dudda

**Affiliations:** 1 Department of Orthopedic and Trauma Surgery, BG Klinikum Duisburg, University of Duisburg-Essen, Großenbaumer Allee 250, 47249 Duisburg, Germany; 2 Department of Trauma, Hand and Reconstructive Surgery, University Hospital Essen, University of Duisburg-Essen, Essen, Germany; 3 Institute for Research in Operative Medicine (IFOM), University of Witten/Herdecke, Cologne, Germany

**Keywords:** Calcaneal fracture, Sinus tarsi approach, Extensile lateral approach, Calcaneal nail

## Abstract

**Background:**

Locking plate osteosynthesis via an L-shaped lateral approach is the gold standard in treating displaced intra-articular calcaneal fractures. High complication rates are known for this approach. The most frequent complications are wound edge necrosis and superficial wound infections. To reduce complication rates, a locking intramedullary nail (C-Nail) was developed that can be implanted minimally invasively via a sinus tarsi approach.

We compared the postoperative complication rate and the outcome of plate osteosynthesis versus C-Nail in displaced intra-articular calcaneal fractures.

**Methods:**

All patients with calcaneal fractures who received osteosynthesis with either plate or C-Nail between January 2016 and October 2019 in our institution were retrospectively analyzed. A subgroup analysis was performed with matched pairs (matching Sanders type, age, Böhler’s angle postoperative in normal range, 33 pairs). Endpoints were postoperative complication rate, bone healing, full weight-bearing and functional outcome. Treatment groups were compared using Fisher’s exact test for binary data, and Mann-Whitney *U*-test for continuous data. A *p*-value < 0.05 was considered statistically significant.

**Results:**

One hundred and one calcaneal fractures were included (C-Nail *n* = 52, plate *n* = 49). Patients with C-Nail developed significantly less postoperative complications (*p* = 0.008), especially wound edge necrosis (*p* < 0.001). Screw malposition was found more often in the C-Nail group. The rates of achieving full weight-bearing as well as bone healing were comparable in both groups, but in each case significant faster in the C-nail subgroup. The results of the matched-pairs analysis were comparable.

**Conclusions:**

The postoperative complication rate was significantly lower in the C-Nail group. The C-Nail appears to be a successful alternative in the treatment of calcaneal fractures, even in Sanders IV fractures because of the minimal-invasive implantation as well as the high primary stability. Long-term analysis of this new implant including elaboration on functional outcome is planned.

**Trial registration:**

Deutsches Register Klinischer Studien (DRKS) DRKS00020395. Date of registration 3 January 2020.

## Background

The surgical treatment of displaced intra-articular calcaneal fractures remains challenging. It aims at the most anatomic reduction possible while restoring joint congruence as well as height, length, and width of the calcaneus [1–3]. Thereby typical long-term consequences such as pain and weight-bearing insufficiency due to post-traumatic subtalar arthrosis up to subtalar arthrodesis shall be prevented or at least diminished [4].

Depending on the conditions of the soft tissue and the type of fracture, various therapeutic procedures are available for calcaneal fractures: conservative treatment [5, 6], open reduction and (locking) plate osteosynthesis [7–9], percutaneous respectively minimal-invasive reduction and plate, screw or K-wire osteosynthesis [3, 7, 9–11], arthroscopically assisted reduction [8, 12], and treatment with external fixator [13] up to primary subtalar arthrodesis, in particular in case of multi-fragmentary calcaneal fractures [14].

The gold standard for displaced intra-articular calcaneal fractures is open reduction and plate osteosynthesis via an L-shaped lateral approach [7, 9, 13, 15–17]. However, high complication rates of up to 47.4% are known for this approach [4, 7, 9, 16–20]. The most frequent complications are wound edge necrosis and superficial wound infections, but deep wound infections/osteomyelitis are also regularly reported.

A sinus tarsi approach causes less complications than an L-shaped lateral approach—particularly with regard to wound edge necrosis and superficial wound infections [4, 11, 19, 21, 22]. The reduction of the posterior facet seems to be comparably good [19, 22, 23].

The C-Nail® was developed to reduce the high complication rate after lateral approach. It is a locking nail that can be implanted minimally invasively [24–26]. The reduction of the subtalar articular surface is performed via the abovementioned sinus tarsi approach. The initial results are promising. To date, however, there have been only a few clinical studies on this new implant [1, 16, 24]. Randomized studies as well as long-term results are lacking.

This retrospective study compares the (early) postoperative complication rate as well as the outcome of C-Nail® versus plate osteosynthesis in displaced intra-articular calcaneal fractures.

We hypothesized that using the C-nail in patients with displaced intra-articular calcaneal fractures results in less early-postoperative complications with comparable long-term results when compared to plate osteosynthesis.

## Methods

### C-Nail

The C-Nail® (manufacturer: Medin, Neustadt in Moravia, Czech Republic; distribution in Germany by tantum AG, Neumünster) is a locking nail made of implant steel. It has a length of 65 mm and a diameter of 8 mm. The nail can be extended with endcaps from 5 to 20 mm to a maximum of 85 mm.

The main tuberosity fragment can be reduced toward the sustentacular fragment percutaneously by a Schanz screw. By applying traction and valgus or varus stress to the Schanz screw, shortening, and varus/valgus malposition of the tuberosity fragment can be corrected. Temporary fixation of the position of the tuberosity fragment is performed with Kirschner wires. A sinus tarsi approach is used for open reduction and control of articular reduction. After reduction, the posterior facet is stabilized with two screws. The joint block and the anterior process are repositioned to the tuberosity and are temporarily fixed with Kirschner wires. Not till then a vertical incision below the attachment of the Achilles tendon is made and a guidewire is placed toward the center of the calcaneocuboid joint. After that, the C-nail is placed. Kirschner wires and screws can be implanted with an aiming device. Up to seven screws can be inserted to stabilize even multi-fragmentary fractures. Finally, the aiming device is removed and an end cap is applied.

The skills of this implant are the minimal-invasive reduction and implantation as well as the reduction via the less complicative sinus tarsi approach. The pitfalls are a limited view to the articular surface due to the sinus tarsi approach, the required reduction before introducing the nail and last but not least a learning curve.

According to the manufacturer, indications are displaced intra-articular fractures (type Sanders I-IV) and unstable extra-articular two-part fractures [16]. Contraindications are unsealed apophyseal joints, infected soft tissue, and calcanei with a length of < 65mm [16, 26].

Since the introduction of the C-nail® in our institution in July 2016, open reduction and plate osteosynthesis are performed less and less frequently. The decision as to which osteosynthesis method is used is determined individually by surgeons’ preference. In order to obtain comparably large groups, patients with displaced intra-articular calcaneal fractures are evaluated from January 2016 onwards.

### Patient cohort

A retrospective analysis was conducted and included patients with displaced intra-articular calcaneal fractures, who were treated in our institution between January 2016 and October 2019 either with C-Nail® or with plate osteosynthesis. Inclusion criteria were at least one consultation postoperative, as a general rule 6 weeks postoperatively. Due to the retrospective design of this study, we had no standardized postoperative follow-up protocol. Patients younger than 18 years, treated with another procedure or a conservative approach, and those who did not appear for follow-up at our clinic postoperatively were excluded.

In addition to our main analysis, we performed a subgroup analysis with matched pairs. Matching was done regarding Sanders type, age, and Böhler’s angle postoperative in normal range.

Fracture type and epidemiological data as well as peri- and postoperative clinical courses were evaluated. Follow-up ended by March 2020.

The primary endpoint is the early-postoperative complication rate. Secondary endpoints are reconstruction of the calcaneus, bone healing, achievement of full weight-bearing, and functional outcome. In addition, post-traumatic subtalar arthroses, subtalar arthrodesis, and all complications including late infections, screw malposition, non-unions, and reoperations were evaluated.

Epidemiologic data and the pre-, peri-, and postoperative clinical course were compiled from the digital patient files and processed pseudonymously. Bone healing and assessment of intra-articular surface were quantified by evaluating x-ray or CT scan. Screw malposition was defined as screw positioning in the subtalar joint as well as overlapping the corticalis > 2 mm.

The functional outcome was assessed the earliest 6 months postoperative with the American Orthopaedic Foot and Ankle Society (AOFAS) Ankle-Hindfoot Scale. Ninety-five to 100 points were defined as excellent results, 75–94 points as good results, and 51–74 points as fair results.

Ninety-two patients with 101 calcaneal fractures (C-Nail *n* = 53, plate *n* = 49) were retrospectively evaluated. Thereof, 76 patients with 82 calcaneal fractures had a follow-up > 6 months (C-Nail *n* = 45, plate *n* = 37) and thus the AOFAS score was analyzed in this group.

### Surgical procedure

Surgery was performed by surgeons of the Department of Foot Surgery. This ensures that the surgeons have sufficient expertise with both strategies. Surgeon’s preference was decisive, if both procedures were possible.

The follow-up consisted of evaluating the endpoints in line with the follow-up care in our consultation hours. All patients were postoperatively treated according to our in-house concept. For 6 weeks postoperatively, patients should avoid weight-bearing of their foot, respectively wear an orthosis with partial weight-bearing. After 6 weeks, patients can perform full weight-bearing.

### Statistics

Treatment groups were compared using Fisher’s exact test for binary data, and Mann-Whitney *U*-test for continuous data. For descriptive analysis, the median was given in addition to mean and standard deviation (SD) in case of a skewed distribution. A *p*-value < 0.05 was considered statistically significant. Statistical analysis was performed using SPSS (Version 24, IBM Inc., Armonk, NY, USA).

## Results

**Fig. 1:**
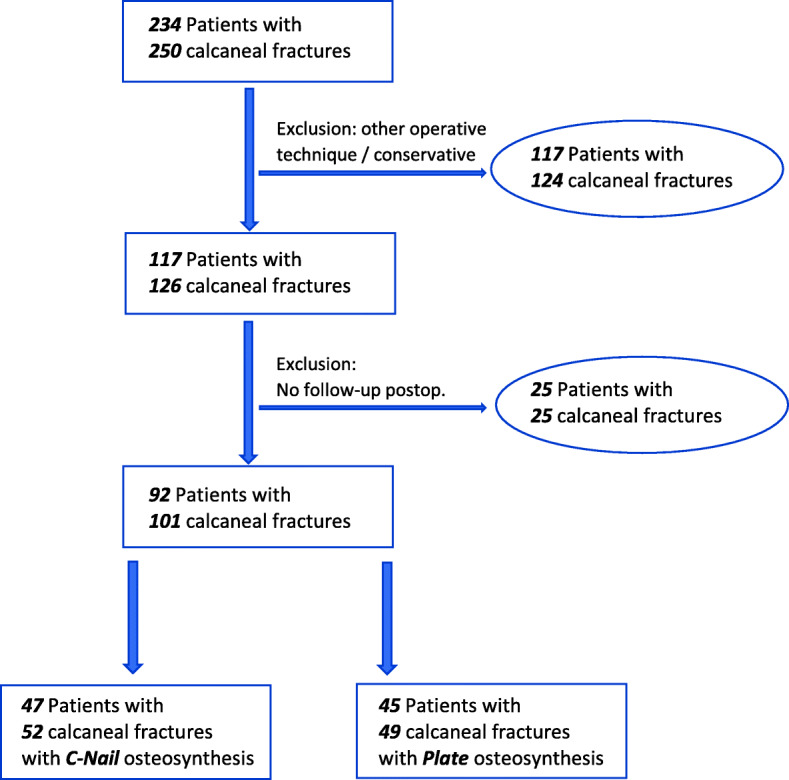
Flowchart of patient inclusion

Ninety-two patients with 101 calcaneal fractures (C-Nail *n* = 52, plate *n* = 49) were retrospectively evaluated (Fig. 1). Thirty-three matched pairs underwent subgroup analysis.

### Epidemiology/preoperative parameters

The two main groups were comparable in terms of sex, type of injury, and type of fracture. However, patients in the C-Nail group were significantly older; patients in the plate group, on the other hand, had a significantly more frequent Sanders IV fracture and a significantly smaller Böhler’s angle preoperatively (Table 1). These differences between groups do not appear in the subgroup with matched pairs.

**Table 1:** Comparison of epidemiologic and preoperative data between C-Nail and plate osteosynthesis

	Total	C-Nail	Plate	Significance (***p***)
		***n*** **= 47 patients**	***n*** **= 45 patients**	
Gender (m)	87 (86%)	44 (84%)	43 (87%)	0.78
Age (years)	46.6 ± 12.1	49.2 ± 13.1	43.9 ± 10.4	**0.019**
		***n*** **= 52 fractures**	***n*** **= 49 fractures**	
Trauma (unilateral, bilateral, multiple trauma in stable condition)	59/22/20 (58%/22%/20%)	29/13/10 (56%/25%/19%)	30/9/10 (61%/18%/21%)	0.72
Type of fracture (open)	6 (6%)	2 (4%)	4 (8%)	0.43
Classification (Sanders type IV)	72 (71%)	32 (62%)	40 (82%)	**0.026**
Böhler’s angle preoperative (median)	16.0 ± 10.0 (15.0)	17.7 ± 9.3 (17.5)	14.2 ± 10.4 (13.0)	**0.028**

The following results refer to the main groups. The results of the matched-pairs subgroup analysis are described at the end of the chapter.

### Perioperative parameters

The average duration of surgery was significantly shorter when using C-Nail. Postoperatively, the Böhler’s angle was within the normal range in 85 patients, more frequently in the C-Nail group, but not significantly. Overall, the average Böhler’s angle was significantly smaller postoperatively in the plate group, whereas the average surgical correction of the Böhler’s angle was comparable between both groups (Table 2). No loss of reduction was found in any group during follow-up.

**Table 2:** Comparison of peri- and postoperative data between C-Nail and plate osteosynthesis

	Total, ***n*** = 101	C-Nail, ***n*** = 52	Plate, ***n*** = 49	Significance (***p***)
**Perioperative data**				
Time between accident and operation (days)	11.6 ± 5.5	11.7 ± 6.4	11.6 ± 4.5	0.63
Hospital stay (days), median	15.7 ± 18.2, 9	13.2 ± 12.4, 9	18.3 ± 22.6, 10	0.49
Duration of operation (min), median	108.1 ± 32.1, 101	98.2 ± 27.8, 93	118.6 ± 33.2, 112	**0.001**
CT postoperative performed (*n*)	72 (71%)	38 (73%)	34 (69%)	0.83
Böhler’s angle postoperative, median	26.9 ± 6.5, 28	28.3 ± 6.0, 28	25.5 ± 6.7, 26	**0.039**
Böhler’s angle postoperative in normal range (*n*)	85 (84%)	47 (90%)	38 (78%)	0.10
Böhler’s angle difference post-preop., median	10.9 ± 8.8, 11.0	10.6 ± 8.3, 9.5	11.3 ± 9.4, 11.0	0.30
**Postoperative data**				
Time duration of follow-up (months)	14.2 ± 12.3	14.0 ± 8.9	14.5 ± 15.2	0.22
Full weight-bearing achieved (*n*)	89 (88%)	49 (94%)	40 (82%)	0.07
Time duration to full weight-bearing (weeks), median	14.0 ± 7.2, 12	12.9 ± 8.5, 10	15.4 ± 5.0, 14	**< 0.001**
Bone healing achieved	85 (84%)	45 (87%)	40 (82%)	0.59
Time duration to bone healing (weeks), median	15.6 ± 11.3, 13	14.3 ± 13.2, 11	17.1 ± 8.7, 14	**0.002**
Shoe inlay, orthopedic shoe	20 (22%)/42 (47%)	11 (22%)/21 (43%)	9 (22%)/21 (51%)	0.68
Planned implant removal	33 (38%)	17 (35%)	16 (42%)	0.66
Subtalar arthrosis	30 (37%)	12 (27%)	18 (49%)	0.07
Subtalar arthrodesis	6 (7%)	2 (4%)	4 (11%)	0.40

### Follow-up

A total of 89 patients reached full weight-bearing during follow-up, significantly faster in the C-Nail group (C-Nail 12.9 ± 8.5 weeks, plate 15.4 ± 5.0 weeks, *p* < 0.001). In 85 patients, fracture healing was found by the end of follow-up, also significantly faster in the C-Nail group (Table 2).

Comparable results were found in functional outcome. The sum of the AOFAS ankle-hindfoot score in patients who had a follow-up of at least 6 months was on average 79.4 points without significant differences between the two groups. In the individual items of the AOFAS score, no significant differences between both groups were found either. Thus, the overall functional outcome was good.

### Complications

A total of 38 patients (37.6%) developed complications during follow-up, significantly more frequently in the plate group. In 22 patients, these complications resulted in at least one additional surgery, with approximately the same frequency in both groups.

Wound edge necrosis was significantly more frequent in the plate group, but not all of them were subject to revision. Deep infections with consecutive osteomyelitis of the calcaneus did not differ significantly in frequency between the two groups. Premature implant removal or free tissue transfer due to infection or soft tissue defect were necessary in both groups without significant differences. A screw malposition was found more frequently in the C-Nail group. Non-unions were found equally frequently in both groups (Table 3).

**Table 3:** Comparison of postoperative complications between C-Nail and plate osteosynthesis

	Total, ***n*** = 101	C-Nail, ***n*** = 52	Plate, ***n*** = 49	Significance (***p***)
Total number of complications	38 (38%)	13 (25%)	25 (51%)	**0.008**
Further operations	22 (22%)	12 (23%)	10 (20%)	0.81
Wound edge necrosis	21 (21%)	3 (6%)	18 (37%)	**< 0.001**
Superficial wound infection	16 (16%)	5 (10%)	11 (22%)	0.10
Osteomyelitis	9 (9%)	4 (8%)	5 (10%)	0.74
Infection with one/multiple pathogens	5 (5%)/6 (6%)	2 (4%)/2 (4%)	3 (6%)/4 (8%)	0.28
Malposition of screws	7 (7%)	6 (12%)	1 (2%)	0.11
Local or free tissue transfer	7 (7%)	3 (6%)	4 (8%)	0.71
Early implant removal	11 (11%)	5 (10%)	6 (12%)	0.76
Non-union	7 (8%)	4 (9%)	3 (7%)	1.00

### Matched pairs (*n* = 33 pairs)

Böhler’s angle preoperative as well as Böhler’s angle postoperative was comparable in both groups. The duration of operation was significant shorter in the C-Nail group. The rates of achieving full weight-bearing and bone healing were comparable, but in each case significantly faster in the C-nail subgroup. Patients of the plate subgroup developed significantly more often subtalar arthroses. Complications in general were more often seen in the plate subgroup with significant more wound edge necroses in the plate subgroup, too. Other complications occurred in both subgroups without significant differences (Table 4).

**Table 4:** Subgroup analysis with matched pairs (matching Sanders type, age, and Böhler’s angle postoperative in normal range); comparison of postoperative data and complications between C-Nail and plate osteosynthesis

	Total, ***n*** = 66 (matched pairs ***n*** = 33)	C-Nail, ***n*** = 33 patients	Plate, ***n*** = 33 patients	Significance (***p***)
Age (years)	44.3 ± 9.7	44.8 ± 10.3	43.8 ± 9.3	
Sanders IV	48 (73%)	24 (73%)	24 (73%)	
Böhler’s angle in normal range	62 (94%)	31 (94%)	31 (94%)	
**Preoperative data**				
Type of fracture (open)	3 (5%)	1 (3%)	2 (6%)	0.56
Böhler’s angle preoperative	16.2 ± 10.5	16.9 ± 9.9	15.6 ± 11.2	0.39
**Perioperative data**				
Duration of operation (min)	106.6 ± 28.8	97.2 ± 22.5	116.0 ± 31.4	**0.015**
Böhler’s angle postoperative	27.9 ± 5.4	28.7 ± 5.8	27.1 ± 5.1	0.27
Böhler’s angle difference post-preop.	11.6 ± 9.9	11.8 ± 9.1	11.5 ± 10.8	0.60
**Postoperative data**				
Time duration of follow-up (months)	15.9 ± 13.5	14.3 ± 9.4	17.5 ± 16.6	0.99
Bone healing achieved	60 (91%)	30 (91%)	30 (91%)	1.00
Time duration to bone healing (weeks)	14.0 ± 8.0	10.6 ± 4.4	17.4 ± 9.3	**< 0.001**
Full weight-bearing achieved (*n*)	61 (92%)	31 (94%)	30 (91%)	0.64
Time duration to full weight-bearing (weeks)	12.9 ± 5.4	10.7 ± 4.8	15.2 ± 5.2	**< 0.001**
Subtalar arthrosis	17 (30%)	5 (17%)	12 (43%)	**0.04**
Subtalar arthrodesis	4 (7%)	2 (7%)	2 (7%)	1.00
AOFAS	81 ± 15.4	80 ± 17	81 ± 13.8	0.91
**Complications**				
Total number of complications	21 (32%)	7 (21%)	14 (42%)	0.06
Further operations	12 (18%)	6 (18%)	6 (18%)	1.00
Malposition of screws	4 (6%)	3 (9%)	1 (3%)	0.34
Wound edge necrosis	13 (20%)	2 (6%)	11 (33%)	**0.005**
Superficial wound infection	8 (12%)	2 (6%)	6 (18%)	0.13
Osteomyelitis	4 (6%)	1 (3%)	3 (9%)	0.30
Infection with one/multiple pathogens	1 (2%)/4 (6%)	0 (0%)/1 (3%)	1 (3%)/3 (9%)	0.34
Non-union	2 (3%)	0 (0%)	2 (7%)	0.16

All in all, the results of the subgroup analysis are comparable to those of the main group analysis.

## Discussion

The optimal treatment of displaced intra-articular calcaneal fractures is challenging and still controversial [7, 9, 10, 19, 24, 27, 28]. The development of postoperative wound complications is a major concern in the treatment of calcaneal fractures with extensile approaches [3].

The aim of our study is to compare the (early) postoperative complication rate as well as the outcome of C-Nail® versus plate osteosynthesis in displaced intra-articular calcaneal fractures.

**Fig. 2:**
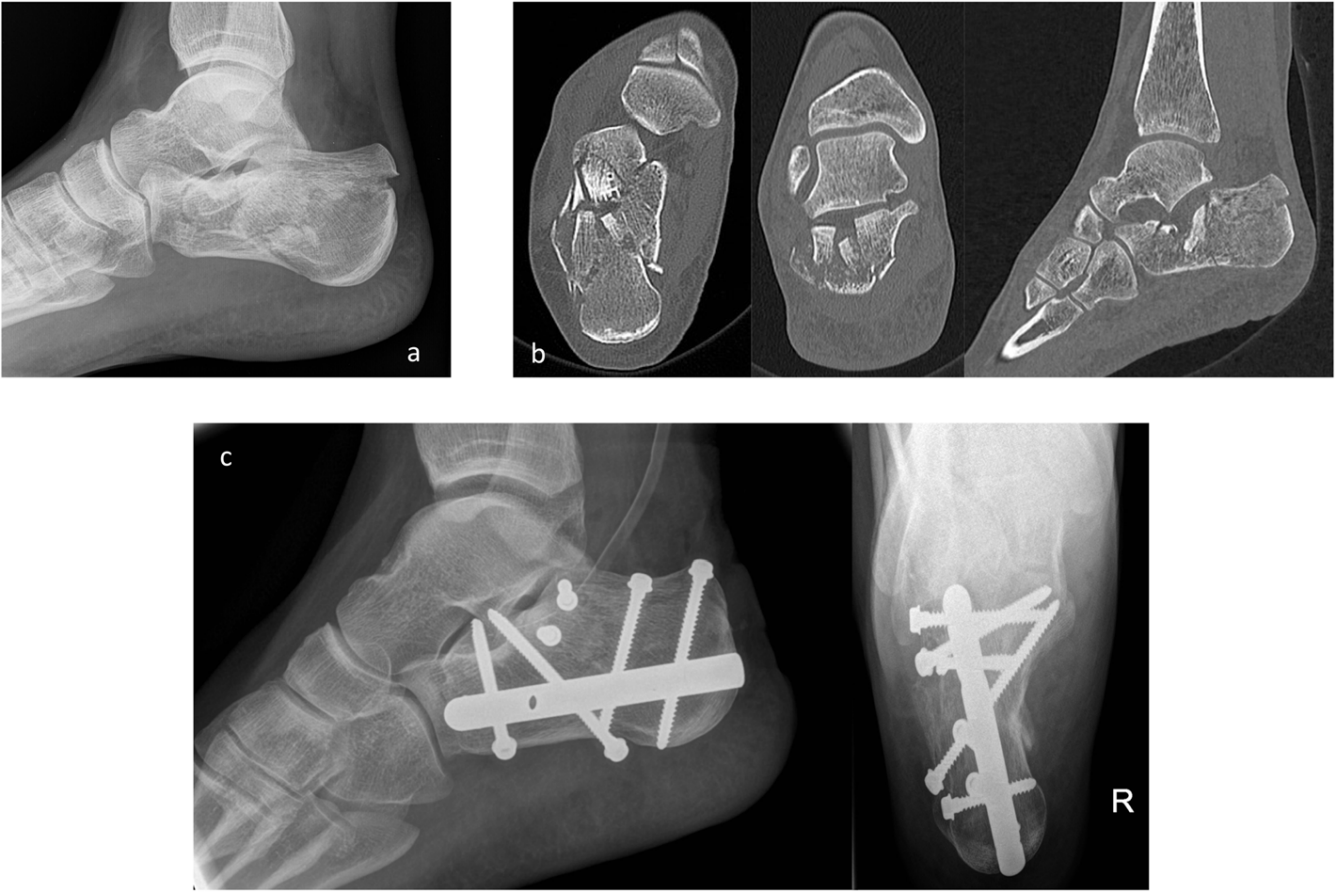
Calcaneal fracture, osteosynthesis with C-nail. **a** Preoperative x-ray. **b** Preoperative CT scan. **c** Osteosynthesis with C-nail, postoperative x-ray

The C-Nail group developed significantly fewer postoperative complications and in particular wound edge necroses, whereas the number of deep infections/osteomyelitis and follow-up operations were comparable. The C-Nail group reached full weight-bearing and bone healing faster (Fig. 2). Comparable results were found in our subgroup analysis with matched pairs.

To date, there have been only few clinical studies on the use of the C-Nail® [1, 16, 24]. So far, only one study by Zeman et al. [1] compared the use of C-Nail® with the gold standard plate osteosynthesis via a lateral approach. Neither the rate of wound edge necrosis nor that of deep infections differs significantly between the groups in this study. The functional outcome for plate and C-Nail® are also comparably good. However, in this study the two groups are of different sizes (plate *n* = 217; C-nail *n* = 19). Furthermore, only Sanders II and III fractures, but no Sanders IV fractures, were treated with C-Nail in this study, so that the comparability to our study is limited. The authors conclude that the C-Nail® can be successfully used as the method of first choice in Sanders type II and III fractures.

Zwipp et al. [24] report on 106 calcaneal fractures that were treated with C-Nail®. A control group is missing. The rate of superficial wound edge necroses and deep infections is low at 1.9% and 0.9%, respectively. Zwipp et al. [24] conclude that the minimally invasive implantation of the C-Nail® with reduction via a sinus tarsi approach leads to low rates of postoperative complications.

Pompach et al. [16] report in their study on the surgical technique of the C-Nail®. They analyzed the same patients as reported in the study of Zwipp et al. [24].

The authors of the latter two clinical studies [16, 24] are patent owners of the C-Nail®, so that a conflict of interest cannot be completely ruled out.

There are numerous studies on the complication rates after osteosynthesis of a calcaneal fracture over an extended lateral approach and further studies comparing these with the complication rates after sinus tarsi approach or other minimally invasive approaches in the current literature [4, 11, 19, 21–23]. In a meta-analysis by Mehta et al. [4], there were significantly fewer postoperative complications after sinus tarsi than after extended lateral access. In general, postoperative wound infection rates after the sinus tarsi approach range between 0 and 9.1% [11, 19, 29–31].

In our study, the early-postoperative complication rate was significantly lower in the C-Nail group than in the plate group with comparably good reduction results. However, not all wound edge necroses or superficial wound infections were subject to revision, many of them could be successfully treated by conservative wound management. It is also notable that the early-postoperative complication rate in the C-Nail group shown in our study—although lower than in the plate group—is nevertheless higher than described in other C-Nail-studies [1, 16, 24]. One explanation may be the higher number of complex fractures, including Sanders IV fractures with often primarily critical soft tissues.

De Groot et al. [28] conclude that short-term postoperative complications do not influence mid- to long-term outcome. The findings of our study do not lead to the same result. Even in conservatively successfully treated wound margin necroses, the duration of rehabilitation was extended with delayed weight-bearing of the affected limb. Thus, no significant difference was found between our groups with regard to the achievement of full weight-bearing and radiological consolidation. However, the C-Nail group—with significantly fewer complications—achieved both full weight-bearing and bone healing significantly faster. Based on our analysis, we assume that postoperative complications negatively influence at least the mid-term outcome.

The duration of surgery is indicated by some authors as a risk factor for postoperative wound healing disorders [4, 9, 32]. In their meta-analysis, Mehta et al. [4] describe a shorter duration of surgery when using the sinus tarsi approach compared to the extended lateral approach. For our patient cohort, both of these factors apply: The patients treated with C-Nail® had a significantly shorter duration of surgery and fewer postoperative wound healing disorders.

According to Court-Brown et al. [33], the deep infection rate correlates with surgical experience. Swords et al. also describe that complications decrease and results improve with surgeons’ experience [34]. A sufficient level of expertise is guaranteed for both groups in our institution since the calcaneal fractures are treated by surgeons who are experienced in foot surgery.

Our analysis noticed an increased rate of screw malposition in the C-Nail group. This complication was found particularly in the initial phase and, in our opinion, it should be seen as sign of a learning curve for a new implant. Amlang et al. [26] also refer to a learning curve in the surgical technique for the use of C-Nails®. We already saw a decrease and expect a future decrease in this respective complication.

Many authors cite the postoperative Böhler’s angle as a relevant factor for the outcome after calcaneal fracture [5, 6, 10, 28, 34, 35]. Su et al. [35] describe a significant correlation with the functional recovery. This opinion is however controversial. Biz et al. [7] conclude that the postoperative Böhler’s angle does not correlate with the clinical outcome. In our study, patients with a postoperative Böhler’s angle < 20°—independent of the group—had a worse outcome measured by the AOFAS ankle-hindfoot scale.

According to Rammelt et al. [12], the most important indicator of prognosis is the postoperative status of the subtalar joint. Other authors consider the meticulous restoration of the subtalar joint congruity to be crucial for the functional outcome [5, 6, 24]. Our results were comparably good in both groups regarding the functional outcome as measured with the AOFAS ankle-hindfoot scale.

Many authors use the AOFAS ankle-hindfoot scale to assess the outcome after calcaneal fractures [5, 7, 9, 10, 19, 22, 28]. Studies report average results in the AOFAS ankle-hindfoot scale between 65 and 85 points [28]. Zwipp et al. [24] report average results of 89.5 points at 6-month and 92.6 points at 12-month follow-up using the C-Nail®. Secondary subtalar arthroses or arthrodeses were not observed. Zeman et al. [1] achieved predominantly excellent and good results with both plate and C-Nail. Our patients achieved an average of 79.4 points at 6-month follow-up and thus good results, but our functional results are somewhat worse than the results of Zwipp et al. [24] and Zeman et al. [1]. However, we have treated significantly more Sanders IV fractures. In the current literature, there is unanimous opinion that Sanders IV fractures have a poorer functional outcome [5, 7, 27]. In relation to the severity of injury in our patients, we consider the functional outcomes to be good.

In summary, there are many factors that can influence the complication rate and the outcome, e.g., the duration of surgery, the surgical experience, the postoperative Böhler’s angle, and the restoration of the subtalar joint.

The minimally invasive implantation of the nail and the reduction of the subtalar articular surface via a sinus tarsi approach appear advantageous.

There are limitations to our study. First of all, it is a retrospective observational study, however with a control group. The two main groups are only comparable to a limited extent, since significant differences in age, fracture classification, and preoperative Böhler’s angle were found. Nevertheless, the results were verified in our subgroup analysis with matched pairs. There is also no standardized follow-up protocol. As a consequence, the level of evidence is weak. The informative value of the AOFAS score is limited due to a follow-up < 12 months in most patients. In general, a longer follow-up would have been desirable to fully assess secondary complications such as subtalar arthroses and arthrodesis as well as functional outcome. The patients will be monitored further. Finally, extrapolation of our results can be difficult, because our patient collective is not representative. Sanders IV is the most common fracture type in our institution. Furthermore, if an orthopedic surgeon has a small number of cases, the learning curve may be flatter. General challenges however, e.g., risk of wound edge necrosis or wound infection, are transferable.

However, in our opinion, this study enlarges the existing knowledge including skills and pitfalls of this implant. The C-nail is a relatively new implant. Most of the existing studies reported of a small patient number. There has been only one comparative study up to now (comparing C-nail with well-established plate osteosynthesis). Our approach showed comparable results to those of previously performed studies. In addition, we demonstrated that the C-nail is an adequate osteosynthesis even in complex fractures, not only in Sanders type II fractures.

## Conclusion

The early-postoperative complication rate was significantly lower in the C-Nail group. Because of the possibility of minimally invasive implantation and the high primary stability, we consider the C-Nail® to be a good and safe implant for the treatment of calcaneal fractures, even in Sanders IV fractures. Long-term analysis of this new implant including elaboration on functional outcome is planned.

## Data Availability

The datasets analyzed during the current study are available from the corresponding author on reasonable request.
